# Community Needs Assessment of Low-Income Sections of 10 Rural Villages in Karnataka and Tamil Nadu, India

**DOI:** 10.7759/cureus.21271

**Published:** 2022-01-15

**Authors:** Tanzim Bhuiya, Krima Thakker, Julia Hyacinthe, Eric Cioe-Pena

**Affiliations:** 1 Medicine, Donald and Barbara Zucker School of Medicine at Hofstra/Northwell, Hempstead, USA; 2 Public Health, Donald and Barbara Zucker School of Medicine at Hofstra/Northwell, Hempstead, USA; 3 Center for Global Health, Northwell Health, New Hyde Park, USA

**Keywords:** rural, public health, global health, india, community needs assessment

## Abstract

Background

India’s health disparities are clearly visible in the southern state of Karnataka. A community needs assessment, one of the first done in this area in over a decade, was conducted to identify unsatisfied needs. The Northwell Center for Global Health worked alongside a local boarding school, Shanti Bhavan, to conduct a needs assessment using the Center for Disease Control and Prevention’s Community Assessment for Public Health Emergency Response tool.

Methods

A community-based cross-sectional survey design was implemented in low-income sections of 10 rural villages in Karnataka throughout February 2019. The target population for this study included people who earned less than US$2 per day. The survey instrument consisted of a questionnaire and tracking form.

Results

One-hundred ninety-seven (197) of 359 households participated in the survey, which encompassed a total of 1,023 individuals. Proper housing structure was the most common need (27.7 %), followed by access to transportation (16.1 %) and access to healthcare (15.2 %). Agitated behavior, sad mood, and frequent worries were the most-experienced behavioral health concerns, with a 47.7%, 41.6%, and 41.1 % prevalence, respectively. Chronic diseases (eg, high blood pressure, diabetes, asthma) were prevalent in 35 of the households (9.7%). The major disease concern in relation to mosquito-borne illness was dengue (36.0 %). Access to healthcare was an issue in 44 of the 197 households (22.3%), with financial reasons being the most common barrier.

Discussion

Notably, there were no expressed needs for basic necessities such as food, water, and medication. This may be due to the help of state programs or a limitation of the survey format. Respondents were most concerned with dengue but are also at risk for other vector-borne diseases, such as malaria and chikungunya, highlighting the need to increase awareness and safety measures. Additionally, mental health problems represent a significant burden of disease.

## Introduction

As the second-most populous country in the world, India makes a substantial contribution to the global burden of disease, accounting for 18% of the world’s deaths [1]. Chronic disease is predicted to account for 53% of all deaths while communicable diseases, maternal health, and nutritional deficits constitute 36% [2]. Within India, there are wide variations in these indicators across gender, caste, education, and geography.

These disparities are exemplified within the southern state of Karnataka. Despite being home to Bangalore, the fifth-most populated city in India, the majority (61.33%) of the population resides in rural areas [3]. The birth rate in Karnataka as of 2016 was 18.5 births per 1,000 population in rural communities versus 16.2 per 1,000 population in urban areas. The death rate in rural communities was 7.9 deaths per 1,000 population compared to 4.9 deaths per 1,000 population in urban areas. Infant mortality also differs at 27 deaths per 1,000 live births in rural areas and 19 deaths per 1,000 live births in urban areas [4]. Life expectancy for females is 71.1 years and 67.1 for males [5].

The public health system in India aims to provide universal access to free healthcare. In 2005, the National Rural Health Mission (NRHM) was launched to strengthen the primary health care system. Investments in the NRHM have improved access and coverage in public health facilities. However, diagnostic services are still mostly unavailable and usually need to be paid for out of pocket [2]. This hinders evidence-based care and the delivery of essential and universal healthcare. Karnataka’s government also utilizes the panchayat raj system. A panchayat is a group of five elderly leaders elected by community members. The system is an installation of local self-government at the village, block, and district levels. Despite national health initiatives and local self-governance, public health measures have not been implemented in many villages, which has perpetuated disparities in health care [6]. It is within this context that a community needs assessment was conducted for a group of villages in Karnataka and the neighboring state of Tamil Nadu.

A community needs assessment is a systematic approach to determine the unsatisfied healthcare needs of a population, with the goal of making changes and improvements to meet these unfulfilled needs. The methodology incorporates qualitative and epidemiological approaches to identify what changes can be afforded [7]. The Community Assessment of Public Health Emergency Response (CASPER) is a tool created by the Centers for Disease Control and Prevention (CDC) to allow public health practitioners and emergency management officials to rapidly determine the health status and basic needs of affected communities. Although it was developed for use in a disaster response scenario, CASPER can be utilized whenever the public health needs of a community are not well-known. The tool is not intended to provide direct services to the affected community, but instead makes use of household-based information to provide a quick, reliable, and accurate assessment of a community’s needs [8]. Due to its efficiency, the CASPER tool was used for this study.

The Northwell Global Health team worked alongside the local boarding school, Shanti Bhavan, which is located southeast of Bangalore city on the border of Karnataka and Tamil Nadu. Shanti Bhavan is a non-profit organization that seeks to educate students and lift them out of generational poverty. The children recruited by the school are from families that earn less than US$2 a day and belong to the former Dalit caste, formerly known as “untouchables” [9]. Former caste positions in India have a clear relationship with current economic status and well-being, where the former lower castes are the least-paid, making socioeconomic mobility extremely difficult [10]. Shanti Bhavan invited Northwell Health with the intention of starting a long-term health partnership, and the two groups decided that a needs assessment was an efficient way to identify the specific health needs of surrounding community members. As a result, the CASPER tool was used to conduct a health needs assessment of low-income sections of 10 rural villages that surround Shanti Bhavan, from which the school recruits employees and students.

## Materials and methods

Study design

A community-based, cross-sectional design was implemented by utilizing the CDC CASPER tool to conduct a rapid needs assessment in the villages surrounding Shanti Bhavan outside of Bangalore, India, in February 2019. This assessment focused on household, social, behavioral, and health needs, along with accessibility needs for basic resources such as water, food supply, and medications (Appendix A). The project was approved by the Institutional Review Boards at Hofstra University in New York and Monk Prayogshala, a non-profit academic research institution based in India. Additional ethical approvals were obtained from the panchayats of each of the villages, as well as the local governments of Karnataka and Tamil Nadu. Oral consent was received from participants due to low literacy levels in the villages surveyed and was documented using the survey tracking form. To ensure consistency and clarity, a consent script was used before the start of each encounter (Appendix B).

Study site

**Figure 1:**
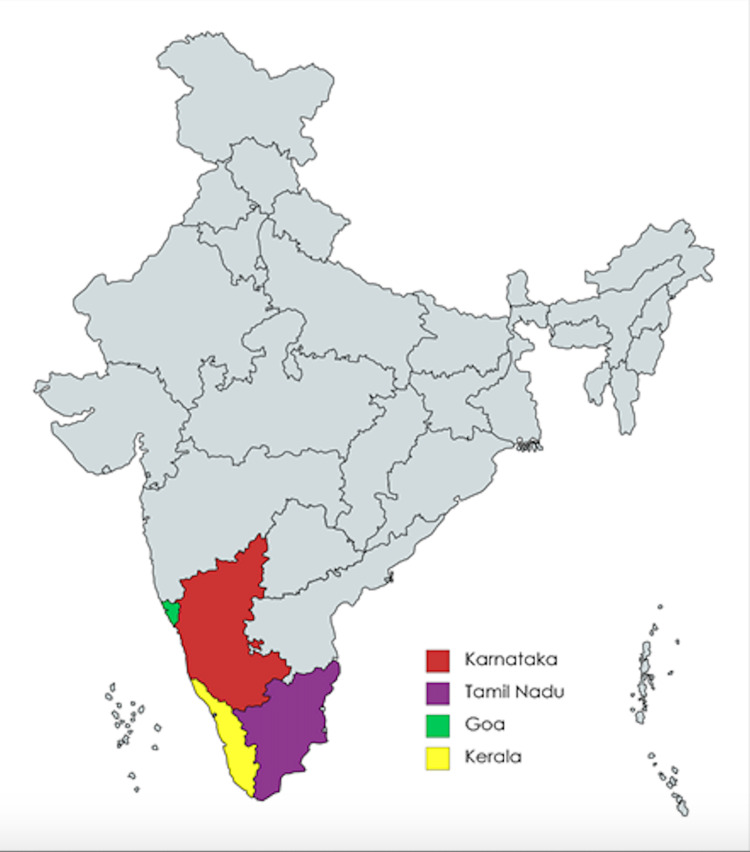
Map of Karnataka, Tamil Nadu, Goa, and Kerala Credit: GlobalData Contract Service Provider Database (Date accessed: 7 November 2021)

The study was conducted by Northwell Health in collaboration with Shanti Bhavan Children’s Project and Baldev Medical and Community center, located in Tamil Nadu, India. The study sites included five villages each from two separate Indian states: Karnataka and Tamil Nadu, which are listed in Table 1. Figure 1 shows the geographic relationship between these two states. These villages were selected based on their proximity to Shanti Bhavan and are areas where the school recruits students and employees.

**Table 1:** Cluster distribution on geographic location

Tamil Nadu, India	Karnataka, India
Alur	Bachahalli
Baliganpalli	Devarapalli
Lakshmipuram	Siddhanahalli
Odapalli	Sonnur
Rajkrishnapuram	Thatanahalli

Study population

The target population for this study included residents of low-income neighborhoods that earned less than $2 US per day. The villages included in the study were geographically segregated by class, with lower-classes living on the opposite sides of the village from upper-classes. Houses were identified for inclusion in the study by their geographical location within the communities and their living conditions. A community liaison helped the survey team to identify eligible houses within the villages. Overall, 359 houses were randomly selected, of which 197 completed the survey.

Inclusion criteria for the study population

The inclusion criteria are 1) families belonging to the section of the village below poverty income status; 2) individuals above the age of consent; 3) self-reported sound state of mind of the interviewee.

Exclusion criteria 

The exclusion criteria are 1) families that belong to the middle or upper classes; 2) families of landlords or the panchayat committee members; 3) individuals below the age of consent.

Instrument

The CDC CASPER tool was used for the rapids needs assessment in this cross-sectional study. This is a validated tool consisting of a specific set of questions designed to provide rapid, inexpensive, and reliable data on the needs of the community based on household public health information [11]. The tool was originally created in English but was translated to the local language of the village (Kannada, Telugu, or Tamil) onsite with the assistance of local volunteer translators from Shanti Bhavan and a community liaison. Translators underwent just-in-time training in advance of data collection where they were taught the objectives, roles, and responsibilities of the team members and the importance of directly translating without paraphrasing. The objective of using this tool was to determine critical health needs by characterizing the population residing in the targeted area. The CASPER toolkit suggests dividing the sampling frame into clusters and recommends the 30x7 sampling design to gain approximately 210 interviews in the sampling frame [12]. We modified the sampling frame to include 10 clusters to engage the ten villages that were included in the study due to a smaller sampling size.

The survey instrument consisted of two components: a questionnaire and a tracking form.

The survey questionnaire consisted of a set of 46 questions, including demographic questions, multiple-choice questions, Likert-type scales, matrix questions, and a few open-ended questions [13]. As per the CDC guidelines, each question had the options of “Don’t Know” and “Refused” [8]. The initial questions of the survey focused on the demographic characteristics of the family by identifying the type of housing structure, the number of family members in the household, and their age groups. The remainder of the questions focused on the social, medical, and behavioral needs of the household (Appendix A).

The tracking form was used to monitor the outcome of every interview attempt, and it was the basis for calculating the response rates (completion, contact, and cooperation) [11]. This form was coded for all the houses, irrespective of their response type. The tracking form was essential in assessing the housing structures and responses from the households, along with assessing some challenges met during the interview phase (Appendix C).

Procedures

Systematic random sampling was used for this study. Each village surveyed was considered as an individual cluster. These clusters were then further systematically divided based on the income status of the households into lower-income groups and middle and upper-income groups. Only the lower-income groups were qualified to participate in the study. Depending on the size of each village, every other house or every third house was selected for the study. Only individuals from houses selected by the randomization process were surveyed. If no one in the household was home or eligible to be interviewed, the house would be revisited up to three times before being marked as incomplete.

The survey team included eight members, consisting of physicians from Northwell Health, Public Health students from Hofstra University, interpreters, and community liaisons that were divided into four pairs. Three teams conducted the surveys while one team monitored the randomization process. Each survey took approximately 25 minutes to complete, including the translation process. The house conditions and the quality of the survey were measured using the tracking form by the team overseeing the randomization process. Each team was accompanied by a translator to assist with the translation of the survey questions from English to languages spoken in the communities (Kannada, Telugu, or Tamil). Prior to the start of data collection, the translators were trained to administer the survey in an efficient and unbiased manner. A trained nurse at Baldev Community Medical Center played the role of community liaison and worked as a mediator between the survey team and the village community. She communicated with the village panchayats or leaders to seek the necessary permissions to conduct the interviews within these villages.

Data management and storage

All surveys were conducted using a printed paper copy of the CASPER tool, along with the tracking form. These surveys were collected and stored by the team managing the randomization process in a secure location. At the end of the surveying process, the responses were entered in the database of Epi Info 7 (Centers for Disease Control and Prevention, 2008) [11]. The descriptive analysis of the survey was carried out using Epi Info 7, and the original survey forms were aggregated and stored in a secure location in Northwell Health.

## Results

This was a pilot study conducted to identify the primary needs of an unidentified population in Karnataka and Tamil Nadu. Three-hundred fifty-nine (359) households were approached to undertake the survey, of which 197 households consented to participate. Of the 162 households that did not participate, 13 households refused to participate while 149 households did not have anyone present to answer at the time. The average response rate for these villages was 57%. Table 2 shows the individual response rates from each village. The total population residing in the 197 houses surveyed was 1,023 individuals. A descriptive analysis of the survey data was performed using Epi Info 7 software (CDC, Atlanta, Georgia).

**Table 2:** Response rates for the survey responses

Village Name	Households Approached	Surveys Completed	Response Rate
Alur	30	15	50%
Baliganapalli	34	20	59%
Devarapalli	40	24	60%
Lakshmipura	17	11	65%
Odapalli	36	16	44%
Oppachalli	34	20	59%
Rajakrishnapura	59	30	51%
Siddhanahalli	41	27	66%
Sonnur	24	14	58%
Thattanahalli	44	20	45%
Total:	359	197	55%

Demographic evaluation

Table 3 discusses the demographic make-up of the 10 villages. The houses selected belonged to the lower-income class of the villages. There was an almost equal proportion of male (50.1%) and female participants (49.9%). Almost two-thirds of the population was between the ages of 18 and 64, followed by 23.7% of the population falling between the ages of 2 and 17. There was a low prevalence of both infant and geriatric populations in these villages, which was believed to be attributable to the poor quality of life and lack of healthcare resources, as stated by the local population. The most prevalent language spoken was Telugu (65.0%), with Kannada, the regional language of Karnataka, as the second most-spoken language (30.5%). A total of 19 deaths were recorded in the 197 houses in the previous year. Of these 19 deaths, 13 individuals were over age 65.

**Table 3:** Demographic variables of the households

Demographic variable	Total population (N)	n	%
Gender -	1,023		
Male		513	50.1
Female		510	49.9
Age groups -	1,023		
Less than 2 years of age		29	2.8
2 to 17 years of age		242	23.7
18 to 64 years of age		665	65.0
Above 65 years of age		68	6.6
Household Languages -	197		
Telugu		128	65.0
Kannada		60	30.5
Tamil		9	4.6
Births and Deaths	1,023		
Birth Rate in the last year		29	2.83
Death Rate in the last year		19	1.86

Housing structures and need

Table 4 demonstrates the housing demographic information from the 10 clusters. The most common housing structures identified within this population were single-family houses (78.7%). This means most of the households lived as nuclear families, as opposed to following a joint family housing system. These houses were evaluated as either intact (none or minimal damage), damaged, or destroyed housing structures using the CASPER tracking form. As per this evaluation, 43 (21.8%) houses were damaged, and nine (4.6%) houses were reported destroyed. Of the 197 households, 117 (59.4%) stated that the most common source of drinking water was tap water. A majority of the households had access to functioning toilets (69.5%). Almost all houses had access to a telephone (91.9%), which proved to be the basic mode of communication. There were a few stated needs for basic necessities such as food, water, and medication. An open-ended question asked the households to mention their greatest need at the time of the survey. Of the 197 households, 112 households provided a response, and a need for a proper housing structure was the most common (27.7%), followed by transportation (16.1%) and healthcare (15.2%).

**Table 4:** Housing structures and needs

Housing structures and needs	n	Total Population (N)	%
Need for water	9	197	4.6
Number of destroyed houses	9	197	4.6
Need for food	11	197	5.6
Need for medications	14	197	7.1
Most common need mentioned by the household – House	31	112	15.7
Number of damaged houses	43	197	21.8
Access to the Internet	69	197	35
Most common source of drinking water - Tap	117	197	59.4
Access to a functioning toilet	137	197	69.5
Most common structure - Single family	155	197	78.7
Access to telephone	181	197	91.9

Behavioral health concerns 

Behavioral health was assessed by the survey through a series of multiple-choice questions. The results are listed in Table 5. Agitated behavior, sad mood, and frequent worries were the most experienced behavioral health concerns with a 47.7%, 41.6%, and 41.1% prevalence, respectively. Twenty-three (23) of the 197 households mentioned instances of witnessing violent behaviors or threats. These were mainly observed in cases of disputes among the neighbors. Table 5 illustrates all of the possible recorded behavioral health concerns.

**Table 5:** Behavioral health concerns

Behavioral health concerns (N=197)	n (%)	%
Unusually happy mood	16	8.1
Experienced violent behaviors/threats	17	8.6
Had traumatic experiences	19	9.6
Loss of appetite	22	11.2
Witnessed violent behaviors/threats	23	11.7
Difficulty concentrating	26	13.2
Trouble sleeping	26	13.2
Thoughts about suicide	35	17.7
Difficulty enjoying things	42	21.3
Nightmares	44	22.3
Frequent worries	81	41.1
Sad mood	82	41.6
Agitated behavior	94	47.7

Other health concerns

Lastly, other health concerns are documented in Table 6. Chronic diseases were prevalent in 35 of the households, with hypertension and diabetes being the most commonly identified in this population. Although mental health is believed to be stigmatized in rural parts of India, a few households reported previous mental health issues (n = 7). When asked about concern for mosquito-borne diseases, 84 households reported being somewhat concerned. The major disease concern in relation to mosquito-borne illness was dengue (36.0%). Lack of access to healthcare was an issue detected in 44 of the 197 households, with a lack of money or high cost being the most common reasons.

**Table 6:** Other health concerns within households

Other health concerns	n (%)
Disease (N = 197)	
Chronic illness	35 (17.7%)
Injuries	28 (14.2%)
Hypertension	22 (11.1%)
Diabetes prevalence	13 (6.6%)
Previous mental health issues	7 (3.5%)
Population concerned with mosquito-borne diseases (N=155)	
Somewhat concerned	84 (42.6%)
Most common disease concern - Dengue	71 (36.0%)
Access to healthcare (N = 197)	
Difficulty in accessing healthcare	44 (22.3%)
Lack of money / cost	32 (16.2%)
Lack of transportation	14 (7.1%)

## Discussion

The results offer a cross-sectional perspective regarding the health and needs of 10 villages surrounding the Shanti Bhavan boarding school. The number one identified need was a proper housing structure, with 26.7% of respondents claiming this was their greatest need. According to observations made on the tracking forms, 43 houses were damaged and nine houses were destroyed (14.5% total).

Contrary to expectations, there were no determined needs for basic necessities, such as food, water, and medication. One-hundred seventeen (117) of the 197 households (59.4%) used government-provided tap water as their source of drinking water. This may be due to the success of the Karnataka Rural Water Supply and Sanitation (KRWSS) project. This project was approved in 2001 and continued through 2014. The goal of the project was to improve access to sustainable drinking water in rural areas using the panchayat system [14]. A similar program exists in Tamil Nadu. The Tamil Nadu Water Supply and Drainage Board has rural water supply schemes that are committed to supplying piped water to all rural households by 2024 [15]. In terms of food supply, the Government of India uses a public distribution system (PDS), which provides certain minimum quantities of food grains to the Government of Karnataka to protect low-income groups [16]. The PDS system supplies rice, wheat, sugar, and kerosene to the Government of Karnataka, which then makes use of ration cards that determine how much citizens are entitled to receive [17]. Shanti Bhavan also aids particularly vulnerable families by providing ragi balls, which are a type of non-perishable food rich in protein.

The threat of mosquito-borne diseases concerned 84 of the 197 households (42.6%). A Likert-style question was used to assess the threat of mosquito-borne disease as seen in question 16 in the Survey Questionnaire (S1 Appendix A). A response of “very concerned” or “somewhat concerned” indicated that the threat of mosquito-borne disease did concern household members. The National Vector Borne Disease Control Program is an integral part of India’s National Rural Health mission. It is responsible for controlling the prevalence of vector-borne diseases such as malaria, dengue, Japanese encephalitis (JE), and chikungunya [18]. According to the needs assessment, 71 of the 84 households (84.5%) that were concerned about mosquito-borne diseases were most concerned about dengue. Conversely, only 40 households (47.6%) were concerned about malaria, and 14 were concerned about chikungunya (16.7%). However, there were 9,655 cases of malaria, 6,105 cases of dengue, 1471 cases of chikungunya, and 746 cases of Japanese encephalitis in the state of Karnataka between April 2016 and March 2017 [19]. In 2018, the state of Tamilnadu had 3,758 cases of malaria and 4,468 cases of dengue [20]. The increased concern of dengue fever compared to malaria may be due to the more severe adverse effects of dengue, including dengue hemorrhagic fever, dengue shock syndrome, and acute respiratory distress syndrome. Dengue has been shown to be a leading cause of hospitalization and death among children in tropical countries [21]. It is important to raise awareness of the risks of other mosquito-borne diseases and actions communities can take to stay safe because dengue is not the only mosquito-borne illness the people in Karnataka are at risk of contracting. Cases of both malaria and dengue spike in the monsoon season (May-October) while chikungunya and JE spike in December [19]. If villagers can be made aware of when the diseases are most prevalent, they can take better actions to both avoid and treat these diseases. A cross-sectional study conducted in two districts in Karnataka showed that only 43.1% of people knew that malaria is transmitted through mosquito bites and only 44.6% were aware of at least one preventive measure to take while 60.8% of people had at least one mosquito net [22]. It is highly likely these percentages would be even lower in the low-income sections of villages surveyed in this study. The provision of insecticide-treated mosquito nets has the potential to be an effective method of reducing mosquito-borne illness transmission and mortality.

Behavioral health was also a point of importance in the survey. Mental health needs, which tend to be largely unmet in poorer communities, represented a significant burden of disease. Over 40% of the population experienced agitated behavior, sad moods, or frequent worries. Despite the significant number of people with behavioral health concerns, only 22 households (11.2%) received services from a counselor, religious leader, therapist, or social worker to address their concerns. One-hundred forty-three (143) of the 197 households (72.6%) had access to a form of counseling but believed they had no need for this service. A possible explanation for this observed gap between the availability and utilization of these resources could be the stigmatization of mental health issues within these populations. This bolsters the need for increasing awareness regarding mental health services along with normalizing the use of these resources to alleviate the burden of disease.

Another interesting finding was that, despite the lack of medical providers serving these rural villages, only 22.3% of respondents identified a lack of access to medical care as a major obstacle. In India, 40% of all health workers work in rural areas; however, over 70% of the population lives in rural areas [23]. As a result of this disparity in workforce density between rural and urban areas, it was expected that the rural communities surveyed would consider access to medical care a much more significant need. A possible explanation could be that rural households in India extensively rely on informal medical providers, who lack medical qualifications [24].

A literature search related to health needs assessments in India yielded several small-scale studies. Though these were less systematic than larger-scale community needs assessments, they highlighted specific obstacles and observations regarding the reality of the health needs of specific communities in rural India. They mention the need for access to personal health services, targeted educational programs on chronic and infectious diseases, and bed net interventions [25-26]. The results of our study expand upon the needs of several more rural villages and demonstrate similar needs years after these initial findings. The findings of these health assessments will guide the creation of a permanent clinic with the help of Northwell Health that will serve the rural villages outside of Bangalore.

Limitations

This study has several limitations. The CASPER survey tool asks questions at the personal and household level. There is a shortage of questions at the community level, including the need for better sidewalks, education, public infrastructure, etc. Additionally, there were limitations as a result of language barriers. Our survey tool was in English and we relied upon volunteer translators to help administer the survey. Although employees were trained to administer the survey objectively, a lack of a common translation of the survey in each language may have led to a bias in results. Additionally, there was a crowding effect during some interviews. Since many of the surveys were conducted outside, there were times where other villagers would walk by and observe the interview. This includes family members, immediate neighbors, or other villagers. This may have affected some survey responses, especially for more sensitive questions such as ones regarding mental health.

## Conclusions

The application of the CDC CASPER tool in this community represented one of the first systematic cross-sectional needs assessment surveys done in this area in over a decade. While limited, the survey data provides interesting insight into the changing needs of poor rural communities in Karnataka and Tamil Nadu, India. The findings of this health needs assessment will guide the creation of a permanent clinic that will serve the rural villages outside of Bangalore. It will also be used to inform the design of interventions created by the Shanti Bhavan and Northwell collaboration to address some of the needs. The creation of a permanent clinic will provide residents of the rural villages access to personal health services that will help combat chronic and infectious diseases. Additionally, the results of our study show that it could be worthwhile to collaborate with other non-profit organizations to address structural housing needs and to provide insecticide-treated mosquito nets.

## Figures and Tables

**Figure 2:**
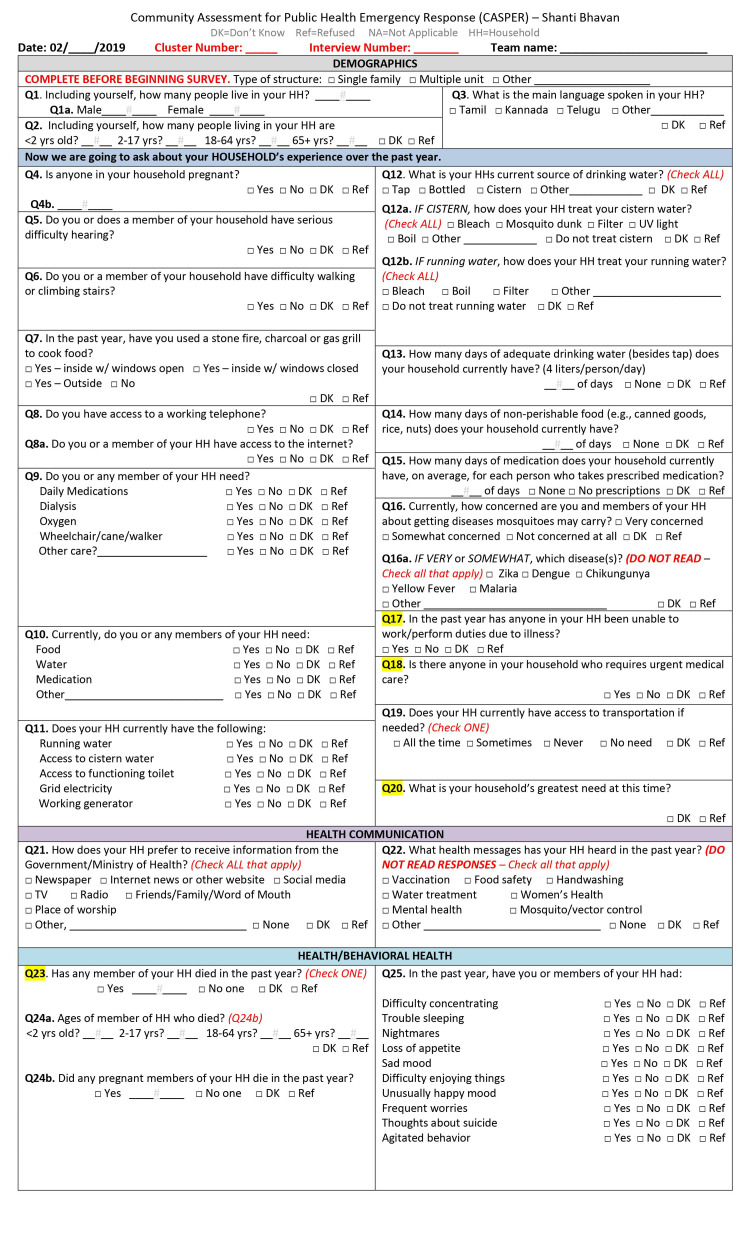
Survey questionnaire

**Figure 3:**
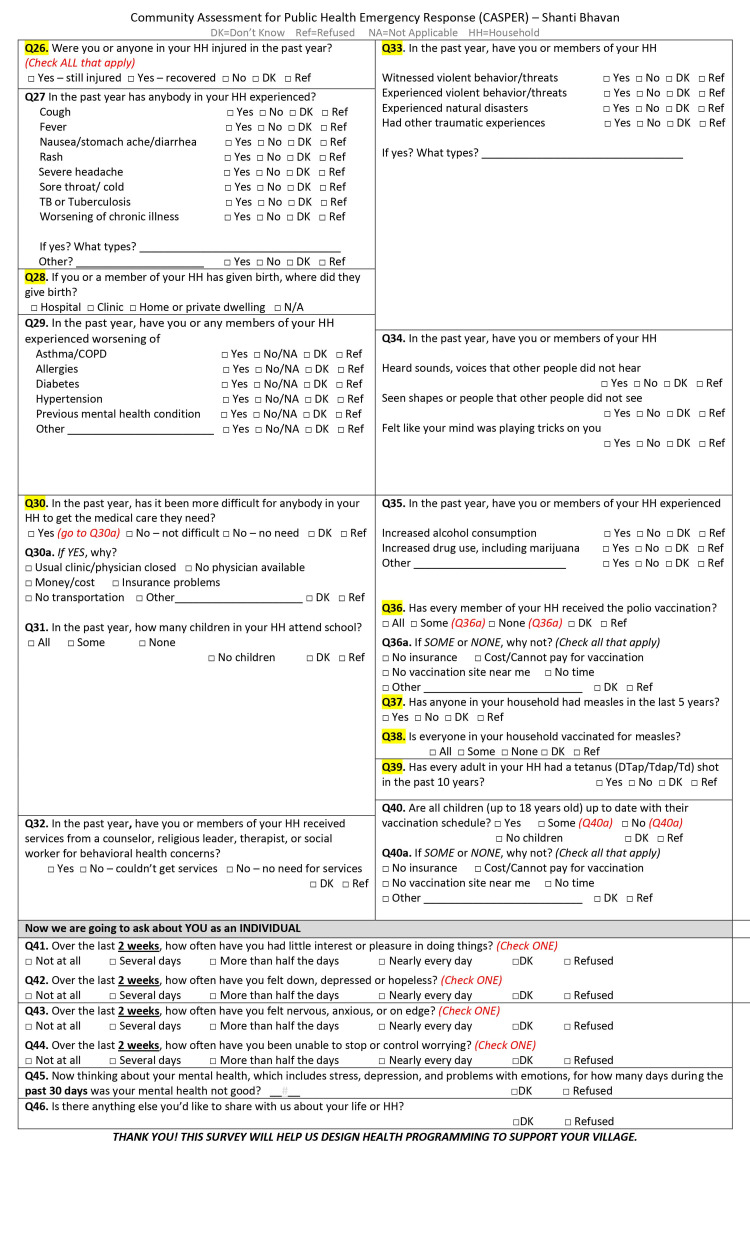
Survey questionnaire back

**Figure 4:**
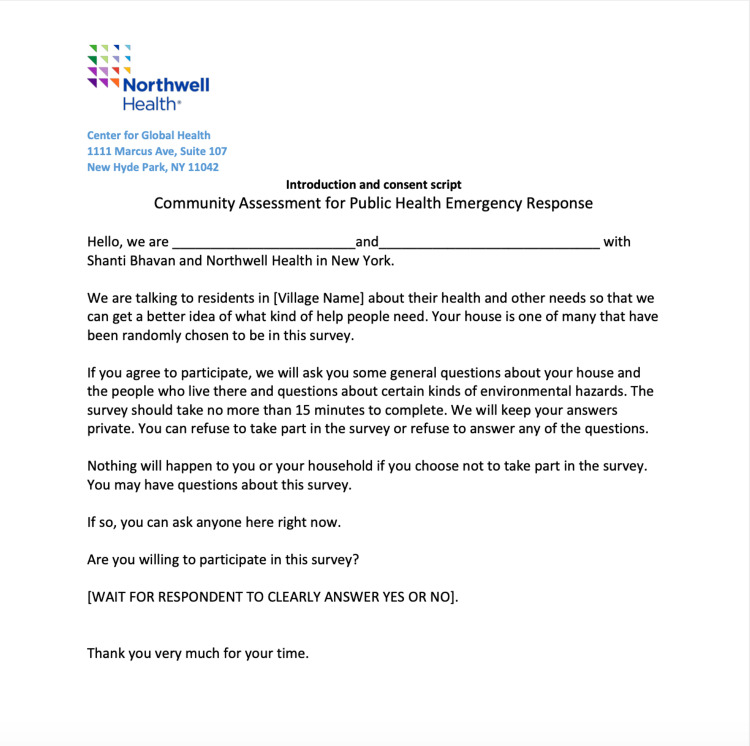
Consent script

**Figure 5:**
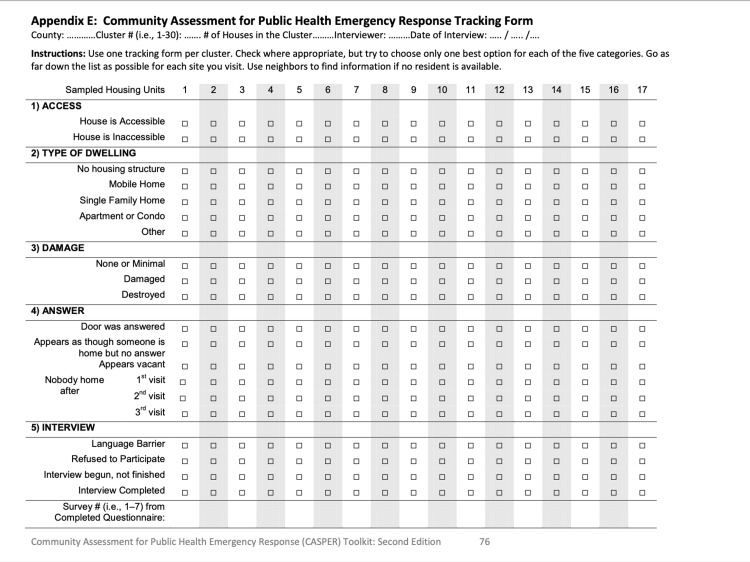
Community assessment for public health emergency response tracking form

## Appendices

Appendix A

Appendix B

Appendix C
